# Antioxidative and Quality Properties of Full-Fat Date Seeds Brew as Influenced by the Roasting Conditions

**DOI:** 10.3390/antiox8070226

**Published:** 2019-07-18

**Authors:** Mohammad Fikry, Yus Aniza Yusof, Alhussein M. Al-Awaadh, Russly Abdul Rahman, Nyuk Ling Chin, Hasanah Mohd Ghazali

**Affiliations:** 1 Department of Process and Food Engineering, Faculty of Engineering, Universiti Putra Malaysia, Serdang, Selangor 43400, Malaysia; 2 Department of Agricultural and Biosystems Engineering, Faculty of Agriculture, Benha University, Moshtohor, Toukh 13736, Qalyoubia Governorate, Egypt; 3 Laboratory of Halal Services, Halal Products Research Institute, Universiti Putra Malaysia, Selangor 43400, Malaysia; 4 Department of Agricultural Engineering, King Saud University, P.O. Box 2460, Riyadh 11451, Saudi Arabia; 5 Department of Food Technology, Faculty of Food Science & Technology, University Putra Malaysia, Serdang, Selangor 43400, Malaysia; 6 Department of Food Science, Faculty of Food Science & Technology, University Putra Malaysia, Serdang, Selangor 43400, Malaysia

**Keywords:** palm date, seeds, brew, roasting process, modelling

## Abstract

Full-fat roasted date seeds are considered an excellent source of antioxidants which can treat many diseases. The specific objectives were to investigate the effect of roasting temperature and time on the hardness of whole seeds, moisture content of the roasted date seeds powder, DPPH radical scavenging activity, total phenolic contents, extraction yield, pH, browning index and sensory properties of the brew prepared from the full-fat roasted date seeds and to construct descriptive models that could describe this effect. Date seeds were roasted at three temperatures (160, 180 and 200 °C) for different period of times (10, 20 and 30 min) using a natural conventional oven; then grinded and next brewed. Hardness of whole seeds, moisture content of the seeds powder, DPPH radical scavenging activity and total phenolic contents, extraction yield, pH and browning index and sensory properties of the brew were significantly affected by the roasting conditions. The statistical results indicated that the proposed model could adequately describe the measured properties. Strong correlations have been found among the properties of the brew as well. The producers of the date seeds brew can utilize these results for controlling the roasting process.

## 1. Introduction 

Antioxidants are substances that are naturally found in fruits, vegetables, legumes and cereals. In foods, they are present in the form of carotenoids and polyphenols or as vitamins. They help to protect human cells from damages as well as aging since they have great curative effects against some diseases (diabetes, hyperlipidemia, obesity hypertension, coronary heart disease, high cholesterol, colorectal and prostate cancers, and intestinal disorders) [1,2,3,4]. They are also useful for preserving food in the form of food additives [2]. These compounds possess various properties including antiradical, anticarcinogenic, antimutagenic and antiproliferative properties [5]. The activity of antioxidants is mostly attributed to their redox properties and their ability to function as reducing agents [5]. Their bioavailability is mainly dependent on their ability to be released during digestion process from food. Thus, determination of antioxidant capacity is helpful for estimating food quality in terms of health [6].

Interestingly, Ghnimi, et al. [7], Bouhlali, et al. [8], Al-Farsi and Lee [9] reported that date seeds are regarded as an important source of antioxidants and phenolic compounds. Remarkably, the antioxidant and total phenolic content of palm date seeds ranged from 65 to 78% [10,11] and from 2697 to 5342 mg gallic acid equivalent/100 g [8], respectively. Previous studies found that the date seeds extract can be effective in scavenging free radicals and in relation to specific diseases [8,12]. As a proof, the date seeds extract caused a noteworthy decline in blood glucose levels in diabetic-compared control rats [13]. For these reasons, palm date seeds could be used as food supplement that helps to treat some chronic diseases, renal stone, bronchial asthma, cough, hyper-activity and weak memory, in declining blood pressure, relaxation of the intestinal and uterine musculature, in growing body protein by reducing fat, in normalizing blood sugar and comforting the pancreas [14,15].

Thus, this waste can be a very useful source to the food processors for developing a new product that contains significant amount of nutrients, especially when the current marketing trend is focusing on natural supplements. Furthermore, discarding of this waste represents an economic wastage. It is particularly apt to produce the decaffeinated substitutes. In Arabian region, the date seeds powder is consumed as a coffee-like drink in the similar way of the Turkish coffee [11,16].

For preparing this kind of brew, roasting is considered the main stage among the production processes. Roasting has many advantages such as promote the aroma and flavour of the beverages and improving the efficiency of post-operations. Roasting deactivates enzymes that can accelerate nutrient loss, terminates undesirable microorganisms and food contaminants, and extends the shelf life of the product. Temperature and time are considered the most important conditions of the roasting process [17]. There are several factors which effect on the optimal required roasting conditions such as, the required roasting level, the type of roaster, cultivar, degree of maturity, and water content of the fresh product. The roasting degree can be observed by the color of the product, by the weight loss, by the taste and odor developed or by the changes in chemical proximate [18]. Recently, antioxidants were extracted from roasted products such as defatted date seeds powder [19], apricot kernel [20], carob powder [21], defatted wheat germ [22] and coffee [23]. However, to the best of the author`s knowledge, limited information has been found on the influence of the roasting temperature and time on the moisture content of the full-fat date seeds powder, the DPPH radical scavenging activity, total phenolic contents, quality and sensory attributes of a brew prepared from the full-fat roasted date seeds. Therefore, this study aimed to explore the effect of roasting conditions on the hardness of whole seeds, moisture content of the full-fat date seeds powder, the DPPH radical scavenging activity, total phenolic contents, quality and sensory attributes of the full-fat roasted date seeds brew and to develop descriptive models for predicting the measured properties under different roasting conditions. 

## 2. Materials and Methods

### 2.1. Preparation of the Powder and Brew

**Figure 1:**
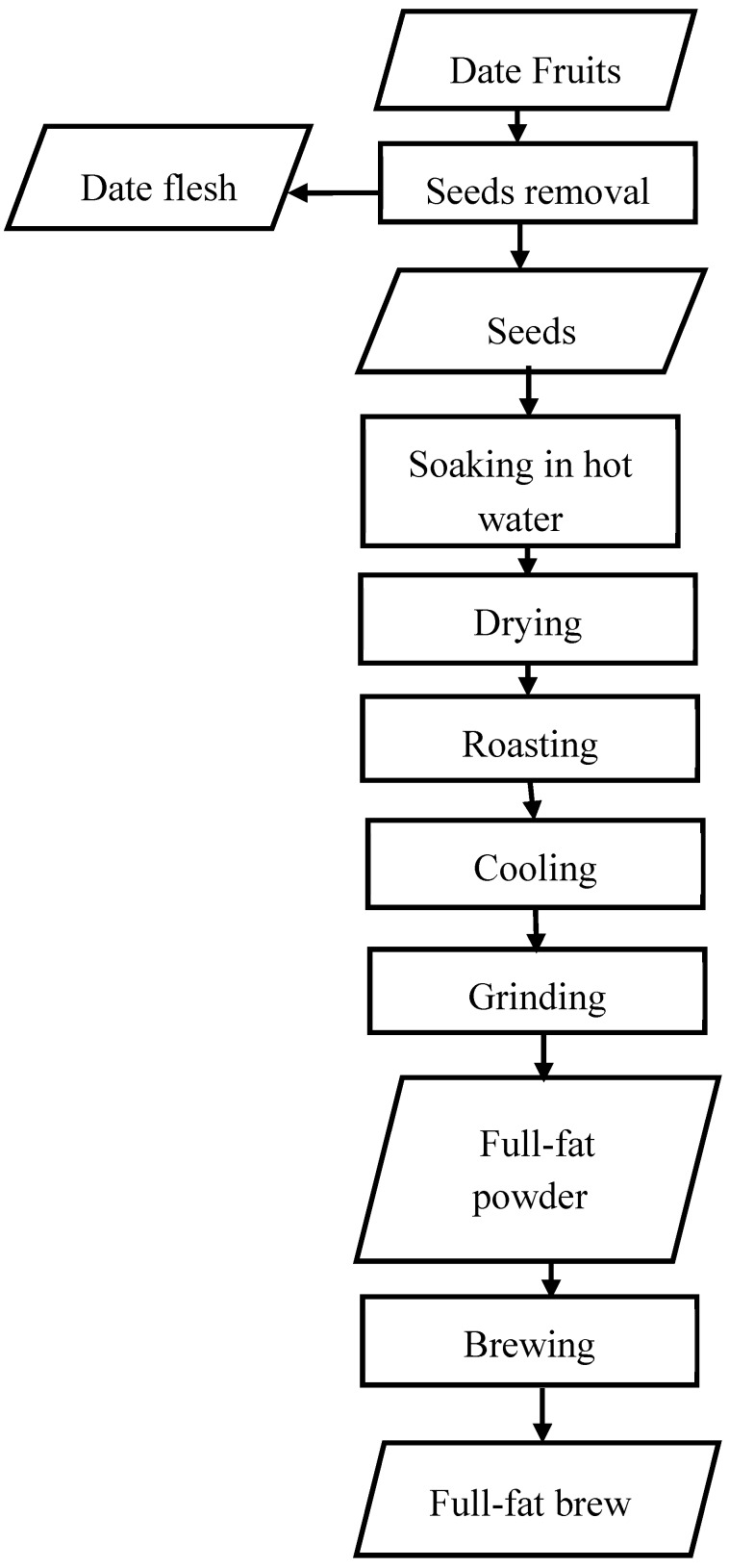
Production flow chart of the brew from the full-fat roasted date seeds.

Date seeds were manually removed from palm date fruit (Sukkari cultivar) which were bought from a well-known market in Riyadh, Saudi Arabia. The seeds (1 kg) were well cleaned by using hot water. Then according the procedure used by Fikry, et al. [19], the seeds were roasted in a laboratory oven (Memmert, UN, Germany) at different roasting temperatures (160, 180 and 200 °C) for 10, 20 and 30 min. These roasting conditions were selected based on those usually used in conventional roasting. To obtain the seeds powder, the roasted seeds were preliminary crushed using a cutting grinder (RT-CR30S, 2007, Taiwan) with 2 mm sieve. The crushed samples were ground by using a hammer mill (Perten, 120, Klaukkala, Finland) equipped with 80-µm sieve. By following the procedure used by Nicoli, et al. [24], the brew was prepared by mixing the powder with a hot water at a ratio of 1:10 *w*/*w*. Then, the mixture was filtered using Whatman No. 2 filter paper (No. 2, ADVANTEC, Tokyo, Japan). Production flow chart of the brew from the full-fat roasted date seeds was drawn in Figure 1. The extracts were stored in glass bottles at −20 °C for further analysis. 

### 2.2. Measurements

#### 2.2.1. Hardness 

According to the method used by Fikry, et al. [17], the hardness of ten roasted date seeds was measured by using Instron Universal Testing 5566 Machine (Instron, Norwood, MA, USA) at 10 mm/min speed. The values of Hardness were derived from the force deformation curve in Newton (maximum beak of first compression) [25]. The data are represented as mean and standard deviation values.

#### 2.2.2. Measurement of Moisture Content

Based on the procedure described in [26] and formerly used by Rahman, et al. [16], the roasted samples (5 g) were dried at 100 °C in a convection oven (Memmert, Schwabach, Germany) until a constant weight was reached. The moisture content (MC% d.b.) was calculated based on the final weight, and the results were reported as the mean of triplicates.

#### 2.2.3. Determination of DPPH Radical Scavenging Activity

To determine the DPPH radical scavenging activity of the date seeds brews, 0.2 mL of the brew 0.8 mL of 0.4 mmol/L DPPH radical were mixed with ethanol. The mixture was powerfully agitated and then left for 10 min. To measure the absorbance of the mixture (Abs), a spectrophotometer (UV1601; Shimadzu, Kyoto, Japan) at 525 nm [27]. Equation (1) was used to calculate the DPPH radical scavenging activity:

(1) $$\mathrm{DPPH}\;\mathrm{scavenging}\;\mathrm{activity}\left(\%\right)=\;\frac{Abs_{\mathrm{control}}-Abs_{\mathrm{sample}}\;}{Abs_{\mathrm{control}}}\times 100$$

#### 2.2.4. Determination of Total Phenolic Contents (TPC)

The total phenolic content (TPC) of the brew was determined by the method of Folin-Ciocalteu reagent (FCR) used by Singleton and Rossi [28]. 5 mL of the brew was added to 5 mL Folin-Ciocalteu reagent in a volumetric flask. Then, after 3 min, 5 mL of 10% Na_2_CO_3_ solution was added, and the mixture was left for 1 h. A spectrophotometer (UV1601; Shimadzu, Kyoto, Japan) was used to determine the absorbance of the mixture at 760 nm. The total concentration of phenolic compounds was calculated by comparison with the absorbance of chlorogenic acid as standard.

#### 2.2.5. Determination of Extraction Yield and pH 

According to the procedure used by Hun-Sik, et al. [29] and Youn and Chung [30], a sample of the date seeds extract (10 mL), was moved into a Petri dish and then dried at 105 °C in a convection oven (Memmert, Schwabach, Germany) until the constant weight of the sample was achieved. The extraction yield was expressed as a ratio of the weight of the extracted solids and the initial weight of the sample. The pH of the date seeds extract was determined by using a Metrohm 654 pH meter with a glass electrode (Metrohm Herisau, Herisau, Switzerland). 

#### 2.2.6. Measurement of Browning Index (BI)

According to the method used formerly by Şahin, et al. [21] and Benjakul, et al. [31] A sample of 15 microliters of the date seed brew were diluted up to 2 mL with demineralized water. Browning index was determined by reading the absorbance of samples at 420 nm, using spectrophotometer (UV1601; Shimadzu, Kyoto, Japan). 

#### 2.2.7. Sensory Analysis of the Brew

According to the procedure used by Fikry, et al. [19], four sensory attributes of the brew samples (color, aroma, taste, and overall preference) were assessed by 30 consuming assessors consisting of Arab people who are familiar with the natural drinks. The sensory assessment was applied in an environmentally controlled room (25 ± 2 °C) under white fluorescent light. A nine-point hedonic scale (1 = disliked extremely; 5 = neither liked nor disliked and 9 = liked extremely) was used by the assessors to evaluate the samples. The samples of date seeds brew were randomly evaluated in white cups, which were coded with random 3-digit numbers, and the samples were evaluated by all the assessors throughout three sessions. Assessors rinsed their mouths between samples using glass of water. 

### 2.3. Statistical Data Analysis

The experiments were designed using a three-level two factor (3^2^) full factorial design (Table 1). Three different roasting temperatures and three different roasting times were considered as independent factors. The dependent responses were the hardness of whole seeds, moisture content of the seeds powder, DPPH radical scavenging activity, total phenolic contents, extraction yield, pH and browning index of the brew. The second order polynomial model (Equation (2)) [32] was used to predict the responses at different conditions.

(2) $$y_{n}=\beta_{0}+\beta_{1}x_{1}+\beta_{2}x_{2}+\beta_{11}{x_{1}}^{2}+\beta_{22}{x_{2}}^{2}+\beta_{12}x_{1}x_{2}$$

where $\beta_{0}$, $\beta_{1}$, $\beta_{2}$, $\beta_{12}$,$\;\beta_{11}$ and $\beta_{22}$ are the model’s coefficients, and $\;x_{1}$ and $x_{2}$ are the predictors (temperatures and times). 

The non-linear regression analysis technique and analysis of variance were used for investigating the effects of the independent variables on the dependent variables. Pearson’s correlation was also run to get the correlation among the quality and chemical attributes. The statistical package for the social sciences (SPSS) (version 21, IBM, New York, NY, USA) was utilized for conducting the statistical analysis. Data were presented as mean ± standard deviation of triplicates.

## 3. Results and Discussion 

### 3.1. Effects of Roasting Conditions on the Physical Properties of the Date Seeds Powder 

Table 2 demonstrates the means and standard deviations values of the hardness of the whole roasted seeds and moisture content (MC % d.b.) of the roasted date seeds powder as affected by the roasting temperature and time.

The hardness could be an indicator of the roasting degree of the date seeds. The hardness of date seeds ranged from 281 N and 2673.9 N at the different roasting temperature and time. The highest roasting temperature and time (200 °C and 30 min) caused the lowest hardness (281 N). Table 2 indicates that there is a significant decrease in the hardness of the roasted whole seeds with increase of the roasting temperature and time. During the roasting process, the date seeds become more brittle as a result of decreasing moisture content and slackening of the structure resulting in the rise of its volume and porosity. Therefore, the roasted seeds at higher temperature require less grinding energy compared to roasted seeds at lower temperature. Similar results were found for *Pistacia terebinthus* beans [33], hazelnut [34], and sesame seeds [35]. 

Table 2 illustrates that the moisture content of the roasted seeds decreased as the roasting temperature and time increase. This result could be attributed to the dehydration of the date seeds during the roasting process. Moisture content of the roasted seeds inclined from 2.29 to 1.47% d.b. A similar trend was found for hazelnut [34] and arabica and robusta coffee beans [18]. 

**Figure 2:**
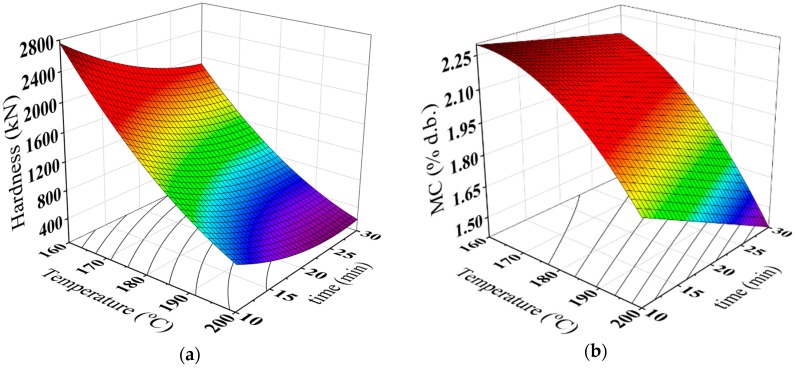
Three-dimensional surface plots for (**a**) hardness of whole seeds, (**b**) moisture content of the full fat roasted date seeds powder.

The results of the analysis of variance for the hardness of the whole seeds, moisture content of roasted date seeds powder are listed in Table 3. It can be observed that there were significant linear and quadratic effects of the roasting temperature and time and their interaction on the hardness, while the moisture content was linearity and quadratically related to the roasting temperature, linearity related to the roasting time. Furthermore, the effect of the interaction between the temperature and time was significant. The surface and contour lines were plotted in Figure 2 for the hardness of the whole seeds, moisture content of roasted date seeds powder. These figures can be satisfactorily used for prediction of the hardness of the whole seeds, moisture content of roasted date seeds powder during the roasting process. Table 4 summarizes the regression coefficients of the second order model (Equation (2)) used for drawing Figure 2a,b.

### 3.2. Effects of Roasting Conditions on DPPH Radical Scavenging Activity and TPC 

The DPPH radical scavenging activity and total phenolic contents can be used for determining the antioxidative activity of the brew under different roasting conditions. Table 2 shows that the DPPH radical scavenging activity increased from around 40 to 81% with increase of roasting temperature and time. These values are comparable with those reported for the roasted date seed extracts [11,19]. 

It can be observed from Table 3 that the DPPH radical scavenging activity of the brew was linearly and quadratically related to roasting temperature. While, the roasting time effected linearly on the DPPH radical scavenging activity of the brew. These findings are close to that reported formerly for coffee brews [23], coffee-like maize beverage [30] and carob extract [21]. 

Recently, the antioxidant activity of the different food materials was found to be increased as the roasting degree increases owing to development of the Maillard reaction products, called melanoidins during the roasting process. The Maillard reaction products are brown-colored compounds with a typical aroma and functional properties including antioxidant activity [23]. The Maillard reaction which is a part of non-enzymatic browning reaction system becomes predominate when components such as reducing sugars and amines (amino acids, peptides or proteins) react with each other during drying or roasting processes. Consequently, roasted foods generally contain numerous levels of Maillard reaction products, which are ideal time–temperature indicators for determining the extent of the roasting process [24,36,37,38,39,40,41,42]. It was reported that development of the Maillard reaction products led to the formation of enediolstructure reductones, which can significantly reduce the oxidation rate of fats leading to increase of DPPH radical scavenging activity [24]. Noteworthy positive relationship between the DPPH and the BI values of the brew were detected (Table 5).

Scalbert and Williamson [43] reported that the phenolic compounds are usually existed in food products and have numerous biological and functional properties that play a vital role in the food quality and human health. Table 2 reveals that the values of the total phenolic content of the brew ranged between 8778.61 and 30,541.39 g/100 mL. The total phenolic contents of the brew increased with increase in roasting temperature and time as it can be seen in Table 2. The increase in the total phenolic contents during roasting could be attributed to the development of Maillard reaction products during roasting [44]. 

**Figure 3:**
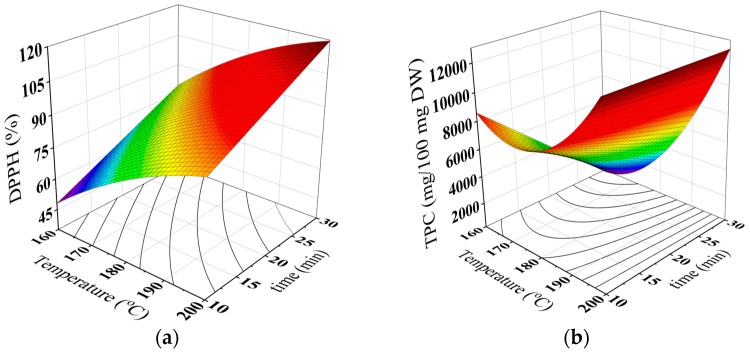
Three-dimensional surface plots for DPPH radical scavenging activity (**a**) and total phenolic contents (**b**) of the brew as a function of roasting temperature and time.

Table 3 illustrated that the TPC of the brew was a function of the roasting temperature with the linear, quadratic and interaction effects. These effects were previously noticed for sesame seeds extract [45], coffee-like maize beverage [30] and carob powder brew [21]. It was suggested that the roasting process could cause evaporation of intracellular water, triggering chemical reactions that may change the lignocellulosic structure and promotes protein denaturation, which may result in a greater availability of the phenolic compounds in the matrix [45]. Also, the increase in the TPC of the brews could be attributed to the formation of Maillard reaction products with phenolic type structure during the roasting process [21,46]. This explanation could be supported by the positive correlation found between BI and TPC (Table 5). Figure 3a,b were plotted using the proposed model (Equation (2)) to describe the changes in DPPH radical scavenging activity and TPC of the brew under different roasting conditions. Table 4 summarizes the regression coefficients of the second−degree polynomial models for the DPPH radical scavenging activity and TPC of the brew.

### 3.3. Effects of Roasting Conditions on the Quality Properties of the Brews

Extraction yield (total soluble solids) was identified as the mass of soluble solids in the brew. Table 2 showed that the maximum extraction yield of the brew was found with a roasting temperature of 200 °C for 30 min. As it can be seen from Table 2, the increase in the roasting temperatures and time caused an increase in the extraction yield of the brew. These results may be associated with the softening of seeds texture for the material flux and the decomposition of insoluble polymers by the roasting temperatures [47,48]. Similar trend was found for maize-like coffee brew [30]. Statistically, the extraction yield has been affected linearly and quadratically by the roasting temperature and it quadratically associated with the roasting time (Table 3). 

Also, Table 2 listed the pH values of the brew as affected by the roasting temperature and time. It can be seen that the pH of the brew decreased with the increase of the roasting temperature and time. The pH values of the brew significantly changed from 5.47 to around 4.60 during roasting period (Table 2). Similar reported were found for carob powder extracts [21,49] and defatted date seed brew [19]. The decrease in the pH value could be explained by the development of acidic caramelization byproducts, such as pyruvic acid, and the formation of Maillard reaction products during the roasting process [49,50]. As shown in Table 5, the significant negative correlation between pH, and BI could support the above explanation.

The formation of non-enzymatic reactions such as the Maillard reaction and sugar caramelization is considered one of browning causes in roasted foods [44,51]. Browning index (BI) can be used as an indicator of the contents of pigment compounds resulted from the non-enzymatic browning reactions [52]. As it can be observed in Table 2, the highest BI value of the brew was detected for a roasting temperature of 200 °C for 30 min. The increase in the roasting temperature and time caused a significant increase in BI (Table 2). A similar trend was found for carob powder extract [21] and coffee brew [23]. 

**Figure 4:**
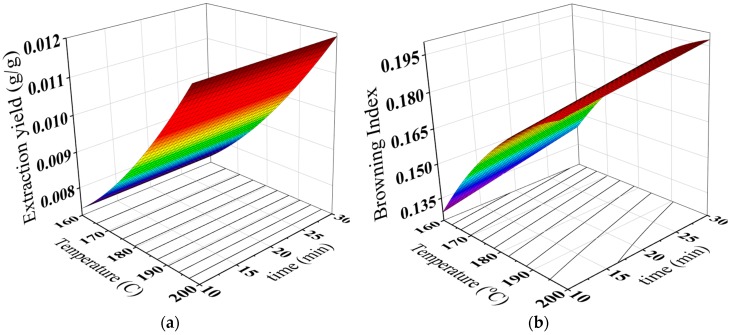

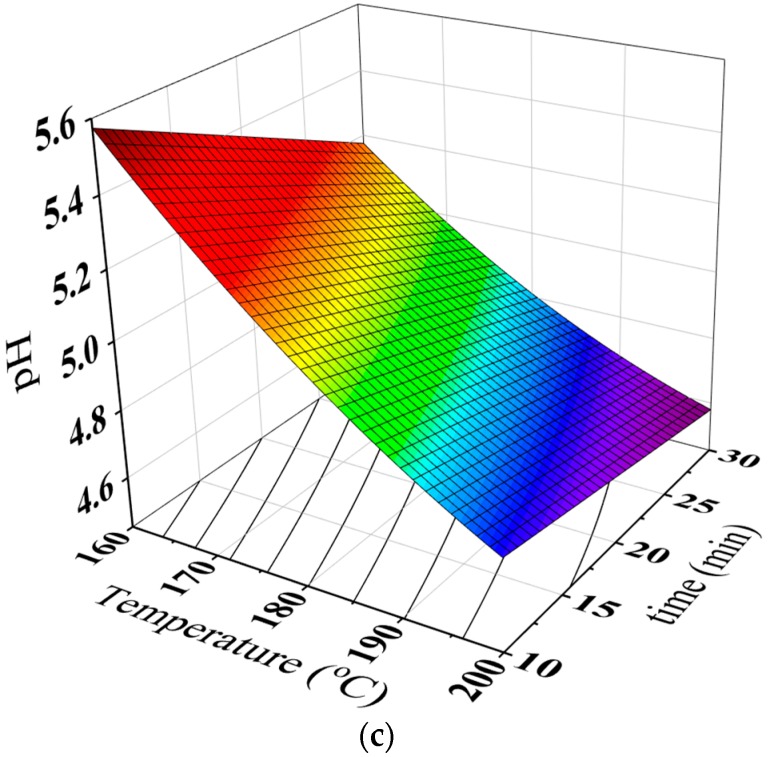
Three-dimensional surface plots for extraction yield (**a**), BI (**b**) and pH (**c**) of the brew as a function of roasting temperature and time.

Statistical analysis showed that the BI of the brew was affected linearly and quadratically by the roasting temperature and time (Table 3). The predicted values of extraction yield, pH and BI can be concluded from Figure 4a–c. Also, the regression coefficients of the proposed models can be found in Table 4. 

### 3.4. Effects of Roasting Conditions on the Sensory Properties of the Brews

Table 6 revealed that the highest color score of the full-fat brew was 6.40 for a roasting temperature of 200 °C and a roasting time of 10 min. Obviously, Table 6 showed that the color score increased and then decreased as the roasting temperature and time increase. As it can be seen from Table 7, the color scores of the full-fat brew were linearly and quadratically related to the roasting temperature and time. Besides, the interaction effect between the temperature and time was found to be significant. It is interesting to observe that the changes in accepting the color of the brew could be due to the increase of BI which was resulted from Maillard reactions [37]. Table 5 indicated that there is a positive relationship between BI and the sensory color of the brew. 

The largest aroma score of the full-fat brew was 5.67 for a roasting temperature of 200 °C and a roasting time of 10 min (Table 6). Noticeably, the aroma scores increased and then decreased with increasing roasting temperature and time (Table 6). It can be seen from Table 7 that the aroma scores were found to be a function of the roasting temperature with linear and quadratic effects, and a function of the roasting time with linear and quadratic effects. The interaction between the roasting temperature and time was also found to be significant. It was suggested that the changes in accepting the aroma could be due to the drop in the pH of the brew. It can be observed from Table 5 that the correlation between the aroma and pH of the brews was found to be negative.

In terms of the taste attribute, it can be observed from Table 6 that the highest score of the full-fat brew was 6.20 obtained at roasting temperature 200 °C and roasting time 10 min. Clearly, taste scores increased and then decreased as the roasting temperature and time increase (Table 6). The taste scores of the full-fat brew were a function of the roasting temperature with linear and quadratic effects (Table 7). The interaction between the roasting temperature and time was found to be significant as well. The changes in accepting the taste could be due to the decline in the pH of the brew that effects on the acid flavor. From Table 5, there is a negative correlation between the pH and the taste of the brew. 

Table 6 indicates that the highest overall preference score of the full-fat brew was 6.53 obtained at roasting temperature 200 °C and roasting time 10 min, respectively. The overall preference scores of the full-fat brew increased and then decreased with the increase of roasting temperature and time (Table 6). Table 7 shows that the overall preference scores the full-fat brew were function of the roasting temperature with linear and quadratic effects. Also, there is a negative correlation between the pH and the overall preference of the brew (Table 5). 

**Figure 5:**
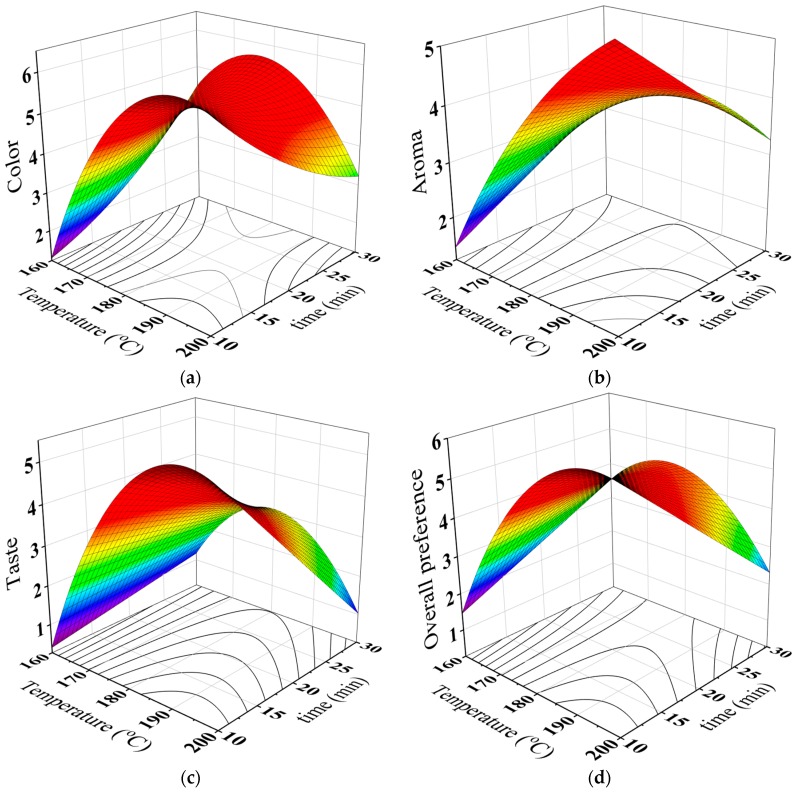
Surface responses and contour plots of color (**a**), aroma (**b**), taste (**c**) and overall preference (**d**) of the full-fat brew from palm date seeds as a function of roasting temperature and time.

Regression coefficients of the predictive models (second degree polynomial) are listed in Table 8. The predicted sensory data were charted in Figure 5.

## 4. Conclusions 

Date seeds can be a good source of the antioxidants that can be used as curative ingredients. The results showed that the roasting temperature and time significantly affected the hardness of the whole date seeds, moisture content of the full-fat roasted date seeds powder, DPPH radical scavenging activity, TPC, extraction yield, pH, browning index and the sensory properties of the brew prepared from the full-fat date seeds. These properties could be satisfactorily predicted by using the second-order model. Strong correlations among the properties of the brew have been found. In addition, brew made from dark date seeds powder was preferred and scored the highest by the panelists in the sensory evaluation. The present results could be utilized to establish indicators for monitoring the quality of full-fat date seeds powder during roasting process and to optimize industrial processing conditions. More detailed investigations are needed to expound the chemical changes which are responsible for the changes in antioxidants during roasting process. Analyzing aroma properties using the electronic nose of the roasted date seeds powder and the impact of adding different levels of normal coffee on the antioxidative properties of the brew from date seeds are also recommended to be investigated in the future. 

## Figures and Tables

**Table 1:** Full factorial design used for the roasting process of the date seeds.

Experiment No.	Coded Factors		Uncoded Factors	
	*x*_1_	*x*_2_	*T* (°C)	*t* (min)
1	−1	−1	160	10
2	−1	0	160	20
3	−1	1	160	30
4	0	−1	180	10
5	0	0	180	20
6	0	1	180	30
7	1	−1	200	10
8	1	0	200	20
9	1	1	200	30

**Table 2:** Means and standard deviations of the physical, antioxidative and quality properties of the whole date seeds, powder and brew as a function of roasting temperature and time.

Roasting Conditions		Hardness (*N*)	MC (% d.b)	DPPH Radical Scavenging Activity (%)	Total Phenolic Contents (mg/100 mg DW)	Extraction Yield (g/g)	pH	Browning Index (Abs at 420 nm)
*T* (°C)	*t* (min)							
160	10	2673.93 ± 45.8	2.29 ± 0.01	40.56 ± 0.13	8778.61 ± 201.6	0.0067 ± 0.0002	5.57 ± 0.06	0.126 ± 0.0004
	20	2226.3 ± 112	2.25 ± 0.02	44.52 ± 0.67	12,718.33 ± 497.1	0.0071 ± 0.0002	5.37 ± 0.15	0.134 ± 0.001
	30	1941.6 ± 83.2	2.21 ± 0.02	47.87 ± 0.80	15,510 ± 631.8	0.0080 ± 0.0001	5.17 ± 0.06	0.142 ± 0.0014
180	10	1667.9 ± 74.3	2.15 ± 0.04	59.49 ± 0.25	11,053.61 ± 432.3	0.0086 ± 0.0004	5.07 ± 0.06	0.163 ± 0.001
	20	901.8 ± 43.5	2.08 ± 0.04	67.68 ± 0.22	13,908.89 ± 1286.6	0.009 ± 0.0002	4.93 ± 0.06	0.172 ± 0.001
	30	756.6 ± 29.9	1.95 ± 0.05	74.64 ± 0.30	14,496.11 ± 568.5	0.0093 ± 0.0002	4.80 ± 0.10	0.175 ± 0.0002
200	10	606.3 ± 11.8	1.80 ± 0.02	78.77 ± 0.07	14,665.28 ± 238.9	0.0106 ± 0.0002	4.70 ± 0.10	0.184 ± 0.001
	20	321.43 ± 68.6	1.58 ± 0.02	80.48 ± 0.80	15,510 ± 631.8	0.0115 ± 0.0001	4.63 ± 0.06	0.186 ± 0.001
	30	281 ± 1.00	1.47 ± 0.06	81.71 ± 0.84	30,541.39 ± 1624.3	0.0121 ± 0.0002	4.57 ± 0.06	0.196 ± 0.003

d.b = dry base, DW= Dry weight.

**Table 3:** Analysis of variance for the physical, antioxidative and quality properties of the whole date seeds, powder and brew.

Source	df ^a^	Sum of Squares						
		Hardness (*N*)	MC (% d.b)	DPPH Radical Scavenging Activity (%)	Total Phenolic Contents (mg/100 mg DW)	Extraction Yield (g/g)	pH	Browning Index (Abs at 420 nm)
Regression model	5	18,430,349 ***	2.147 ***	6317.16 **	783,829,540 **	0.000082 **	2.82 **	0.014777 **
T	1	523,516 ***	0.0796 ***	219.71 **	53,783,177 *	0.000 *	0.05314 **	0.000608 **
t	1	325,687 ***	0.0954 ***	25.68 *	46,245,889 *	0.0000	0.05677 **	0.00003 **
T^2^	1	393,014 ***	0.02098 **	147.11 **	54,003,333 *	0.000001 **	0.02667 *	0.0000 **
t^2^	1	176,233 ***	0.00031	0.89	16,549,281	0.0000	0.000000	0.000428 **
T*t	1	124,237 ***	0.0494 ***	14.3	52,152,801 *	0.0000	0.05333 **	0.000017 **
Error	21	275,381	0.02863	105.81	168,965,129	0.000001	0.12667	0.00007
Lack−of−Fit	3	205,941	0.00763	100.5	156,858,804	0.000	0.000000	0.000048
Pure Error	18	69,440	0.02100	5.31	12,106,325	0.000001	0.12667	0.000023
Total	26	18,705,730	2.17525	6422.97	952,794,669	0.000083	2.94667	0.014848
*R* ^2^		0.985	0.987	0.983	0.823	0.969	0.987	0.957

^a^ degree of freedom; Significance level: *** *p* ≤ 0.001, ** *p* ≤ 0.01, * *p* ≤ 0.05.

**Table 4:** Regression coefficients of the second order polynomial model for physical, antioxidative and quality properties of the whole date seeds, powder and brew.

Model’s Constants	Hardness (*N*)	MC (% d.b)	DPPH Radical Scavenging Activity (%)	Total Phenolic Contents (mg/100 mg DW)	Extraction Yield (g/g)	pH	Browning Index (Abs at 420 nm)
$\beta_{0}$	31,489 ***	−6.23 ***	−525.2 **	253,496 *	0.02132 *	15.1 **	−0.7953 **
$\beta_{1}$	−266.8 ***	0.1041 ***	5.466 **	−2704 *	0.000007	−0.085 **	0.009089 **
$\beta_{11}$	−192.9 ***	0.0446 **	−0.01238 **	7.5 *	−0.00025 **	0.000167 **	−0.00002 **
$\beta_{2}$	0.582 ***	−0.00032 ***	1.56 *	−2093 *	0.000001 **	−0.0733 **	0.001698 **
$\beta_{22}$	1.714 ***	0.000072	−0.00385	16.6	0.0000	0.0000	0.000001
$\beta_{12}$	0.509 ***	−0.00032 ***	−0.00546	10.42 *	0.0000	0.000333 **	−0.000006 *

Significance level: *** *p* ≤ 0.001, ** *p* ≤ 0.01, * *p* ≤ 0.05.

**Table 5:** Pearson’s correlation matrix of the antioxidative and quality characteristics of the brew.

Property	pH	DPPH	TPC	BI	Color	Aroma	Taste	Overall Preference
pH	1							
DPPH	−0.613 **	1						
TPC	−0.666 **	0.399 *	1					
BI	−0.971 **	0.640 **	0.651 **	1				
Color	−0.534 **	0.378	0.020	0.584 **	1			
Aroma	−0.805 **	0.511 **	0.382 *	0.795 **	0.848 **	1		
Taste	−0.651 **	0.475 *	0.090	0.695 **	0.962 **	0.852 **	1	
Overall preference	−0.536 **	0.365	0.057	0.597 **	0.978 **	0.845 **	0.942 **	1

* and ** refer to the significance levels *p* ≤ 0.05 and *p* ≤ 0.01, respectively.

**Table 6:** Means and standard deviations of sensory attributes of coffee-like brew from full-fat roasted date seeds as a function of roasting temperature and time.

Roasting Conditions		Color	Aroma	Taste	Overall Preference
*T* (°C)	*t* (min)				
160	10	1.47 ± 0.30	1.60 ± 0.20	1.13 ± 0.12	1.60 ± 0.20
	20	2.47 ± 0.31	2.67 ± 0.31	1.53 ± 0.42	3.20 ± 0.35
	30	4.46 ± 0.31	4.67 ± 0.31	3.47 ± 0.31	4.67 ± 0.31
180	10	5.27 ± 0.50	4.27 ± 0.31	4.40 ± 0.35	5.33 ± 0.42
	20	5.40 ± 0.35	4.47 ± 0.12	5.53 ± 0.42	5.60 ± 0.20
	30	5.80 ± 0.20	5.07 ± 0.12	5.87 ± 0.12	5.67 ± 0.31
200	10	6.40 ± 0.20	5.67 ± 0.12	6.20 ± 0.01	6.53 ± 0.31
	20	4.26 ± 0.31	5.27 ± 0.58	4.27 ± 0.31	4.53 ± 0.31
	30	3.27 ± 0.12	4.47 ± 0.12	3.20 ± 0.20	3.67 ± 0.12

**Table 7:** Analysis of variance for of the sensory attributes of full-fat brew.

Source	df	Sum of Squares			
		Color	Aroma	Taste	Overall Preference
Regression model	5	63.2889 **	40.0415 **	73.6622 **	54.0211 **
T	1	23.5497 **	3.1126 **	28.4293 **	17.4922 **
t	1	15.8855 **	9.3725 **	12.9295 **	17.5833 **
T^2^	1	18.7267 **	1.7785 **	23.2067 **	13.5 **
t^2^	1	0.96	0.1452	0.4267	0.1067
T*t	1	28.2133 **	13.6533 **	21.3333 **	26.4033 **
Error	21	2.2578	1.8815	6.1244	2.1389
Lack−of−Fit	3	0.5778	0.4681	4.6844	0.6189
Pure Error	18	1.68	1.4133	1.44	1.52
Total	26	65.5467	41.923	79.7867	56.16
*R* ^2^		0.966	0.955	0.923	0.962

* and ** refer to the significance level *p* ≤ 0.05 and *p* ≤ 0.01, respectively.

**Table 8:** Regression coefficients of the second order polynomial equation for the sensory attributes of full-fat brew.

Regression Coefficients ^a^	Color	Aroma	Taste	Overall Preference
$\beta_{0}$	−172.3 **	−68.77 **	−188.7 **	−150.3 **
$\beta_{1}$	1.789 **	0.6506 **	1.966 **	1.5422 **
$\beta_{11}$	−0.004417 **	−0.001361 **	−0.004917 **	−0.00375 **
$\beta_{2}$	1.227 **	0.9422 **	1.1067 **	1.2906 **
$\beta_{22}$	0.004 **	0.001556	0.00267	−0.001333
$\beta_{12}$	−0.007667 **	−0.005333 **	−0.006667 **	−0.007417 *

* and ** refer to the significance level *p* ≤ 0.05 and *p* ≤ 0.01, respectively. ^a^ These are coefficients of Equation (2), and subscripts 1 and 2 represent roasting temperature and roasting time, respectively.
